# Multiparametric MRI radiomics and machine learning for outcome prediction in acute ischemic stroke: a retrospective study of 353 patients

**DOI:** 10.3389/fnins.2026.1843917

**Published:** 2026-10-22

**Authors:** Xiaoxing Peng, Jiannan Guo, Qiuyan Zhou, Shunyu Wang, Tingting Li

**Affiliations:** 1 Department of Medical Imaging, Affiliated Hospital of Changchun University of Chinese Medicine, Changchun, Jilin, China; 2 Department of Electro-diagnosis, Affiliated Hospital of Changchun University of Chinese Medicine, Changchun, Jilin, China; 3 Department of Lung Disease, Tumor and Blood Center, Lung Oncology, Affiliated Hospital of Changchun University of Chinese Medicine, Changchun, Jilin, China; 4 Department of Catheterization Room, Affiliated Hospital of Changchun University of Chinese Medicine, Changchun, Jilin, China; 5 Department of Electro-diagnosis, Qian Wei Hospital of Jilin Province, Changchun, Jilin, China

**Keywords:** acute ischemic stroke, diffusion-weighted imaging, multiparametric MRI, perfusion imaging, prediction model, prognosis

## Abstract

**Background:**

Acute ischemic stroke (AIS) remains a leading cause of death and disability worldwide. Early prediction for clinical outcomes is essential for guiding therapy. Multiparametric Magnetic Resonance Imaging (MRI) offers detailed characterization of ischemic tissue and may predict prognosis in AIS patients. This study assesses the prognostic value of multiparametric MRI parameters in predicting functional outcome, recurrence risk and mortality in AIS patients.

**Methods:**

We conducted a retrospective analysis of 353 AIS patients who underwent multiparametric MRI within 24 h of onset. MRI metrics including diffusion-weighted imaging (DWI) lesion volume, ADC minimum, perfusion parameters and Susceptibility-Weighted Imaging (SWI) microbleeds were correlated with National Institutes of Health Stroke Scale (NIHSS) and modified Rankin Scale (mRS) scores at discharge and 90 days. Predictive performance for clinical outcomes was assessed by receiver-operating characteristic (ROC) analysis.

**Results:**

Among 353 AIS patients with a mean door-to-imaging time of 27 ± 10 min, higher NIHSS, larger DWI volume, prolonged mean transit time (MTT) and Tmax, and increased microbleeds significantly correlated with mortality and disability, while lower ADC, CBF, and CBV correlated inversely with clinical outcomes. NIHSS ≥10.5 and DWI lesion volume ≥52.5 mL yielded AUCs of 0.963 and 0.975 and accuracies of 91.35% and 92.79%, respectively; CBF ≤ 20.5 yielded an AUC of 0.975. NIHSS ≥8.5 and MTT ≥8.5 s showed AUCs of 0.948 and 0.931, respectively, for predicting hemorrhagic transformation. Perfusion thresholds modestly predicted 90-day recurrence (AUC = 0.7). Hypertension, diabetes, and prior stroke were significant clinical predictors of poor outcome. These predictive metrics reflect internal, within-cohort validation only; independent external validation is required before clinical application.

**Conclusion:**

Multiparametric MRI provides robust prognostic information in AIS. Quantitative thresholds for perfusion and diffusion parameters help in risk stratification for hemorrhage and mortality, enhancing individualized management and outcome prediction.

## 1 Introduction

Acute ischemic stroke (AIS) imposes a tremendous global health burden. Stroke is the second leading cause of death and the third leading cause of combined death and disability, with nearly 12 million people experienced a stroke worldwide in 2021 (Feigin et al., 2022, 2025). The estimated global cost of stroke care exceeded $890 billion (Feigin et al., 2025). In the United States alone, roughly 795,000 people experience a stroke annually, about 87% of which are ischemic in origin. AIS is the leading cause of serious long-term disability, reducing mobility and independence in a large proportion of survivors (Stroke, 2025). The high burden of AIS underscores an urgent need for early and accurate diagnosis of stroke for initiation of reperfusion therapies and better clinical outcomes.

The current guidelines recommend rapid neuroimaging in suspected AIS, typically recommending non-contrast Computed Tomography (NCCT) on head as the first-line study to exclude hemorrhage and identify large-vessel occlusions (Powers et al., 2019). However, NCCT has well-known limitations in detecting early ischemic changes. Indeed, NCCT has low sensitivity between 43% and 71% for small infarcts within the first 3-6 h of onset (Gauriau et al., 2023). By contrast, diffusion-weighted Magnetic Resonance Imaging (DWI-MRI) can detect acute ischemia within min of onset with very high sensitivity ranging between 90–97% with subsequent early intervention and better outcomes (Nagaraja, 2021; Fiebach et al., 2002). Multiparametric MRI, which combines multiple sequences such as DWI, perfusion-weighted imaging (PWI), Fluid-attenuated inversion recovery (FLAIR), and MRI angiography, offers much more comprehensive tissue characterization and detection of small infarctions (Osama et al., 2020). DWI is the most sensitive modality for detecting acute ischemic lesions, even in cohorts treated with thrombolysis, with missing only 8% of true strokes who had typically small infarctions with milder symptoms (Simonsen et al., 2015). Adding PWI and FLAIR to DWI further enhances sensitivity of lesion detection with identification of approximately 97.5% of AIS (Simonsen et al., 2015; Tavakkol et al., 2024). Based on these insights, multiparametric MRI could greatly improve diagnostic confidence in AIS and distinguish it from other neurological disorders. It also facilitates measurement of lesion volume, with possible prediction of prognosis (Nael et al., 2017).

Given the high accuracy of multiparametric MRI modalities, the majority of studies focused on traditional CT tools with limited studies assessing the predictive value of multiparametric MRI or combined MRI modalities for clinical outcomes in AIS patients (Quan et al., 2021; Wei et al., 2024). Additionally, recent studies reported the need for understanding the diagnostic and predictive value of combined MRI modalities in acute stroke (Wei et al., 2024; Teitcher and Biller, 2022). Thus, we conducted this study to investigate the role of multiparametric MRI in predicting clinical outcomes in AIS through evaluation of functional disabilities, risk of hemorrhagic transformation, recurrence and mortality outcomes.

## 2 Methods

### 2.1 Study design and population

This retrospective cohort study investigated the prognostic value of multiparametric MRI in patients with acute ischemic stroke (AIS). A total of 353 consecutive adult patients diagnosed with AIS and admitted to the participating center were included. All patients underwent multiparametric MRI within 24 h of symptom onset. Patients aged ≥18 years with confirmed AIS, documented admission National Institutes of Health Stroke Scale (NIHSS) scores, and available modified Rankin Scale (mRS) scores at discharge and at 90 days were eligible for inclusion. Patients with primary intracranial hemorrhage, intracranial tumors, structural brain abnormalities, or incomplete imaging data were excluded. Patients with severe motion artifacts or missing follow-up clinical outcomes were also excluded. This study gained institutional review board (IRB) approval with the number (CCZYFYLL-SQ-2024-224). The IRB waived the requirement for written informed consent because of the retrospective nature of the study. We coded data and characteristics of patients to maintain confidentiality. The study adhered to the Declaration of Helsinki and complies with data protection regulations.

### 2.2 Clinical data collection

Demographic and clinical data were extracted from hospital electronic medical records. Variables included age, sex, smoking status, vascular risk factors (hypertension, diabetes mellitus, atrial fibrillation), prior stroke history, and door-to-imaging time (time from hospital arrival to MRI acquisition under our center's expedited stroke-imaging protocol; this interval was used in place of the full prehospital symptom-onset-to-imaging time, which was not uniformly captured owing to variability in prehospital recognition and transport times). Stroke severity was assessed using the NIHSS at admission. Functional outcomes were evaluated using the modified Rankin Scale at discharge and at 90 days. Poor functional outcome was defined as mRS ≥3. Clinical endpoints included in-hospital mortality, hemorrhagic transformation, and recurrent ischemic stroke within 90 days.

### 2.3 MRI acquisition protocol

All patients underwent standardized multiparametric MRI using a 3-Tesla scanner (MAGNETOM Skyra, Siemens Healthineers, Erlangen, Germany) equipped with an 8-channel head coil. The imaging protocol included diffusion-weighted imaging (DWI), apparent diffusion coefficient (ADC) mapping, perfusion-weighted imaging (PWI), susceptibility-weighted imaging (SWI), and fluid-attenuated inversion recovery (FLAIR).

DWI images were obtained with a b-value of 1,000 s/mm^2^. Perfusion imaging was performed using dynamic susceptibility contrast techniques after intravenous injection of gadolinium contrast. Perfusion maps including cerebral blood flow (CBF), cerebral blood volume (CBV), mean transit time (MTT), and Tmax were generated using standard deconvolution algorithms. FLAIR imaging was acquired using repetition time (TR) of 9,000 ms and echo time (TE) of 140 ms. DWI sequences used TR/TE of 4,200/78 ms with a slice thickness of 5 mm, and SWI was acquired with TR/TE of 27/20 ms, flip angle 15°, and slice thickness 2 mm, in accordance with the vendor's standard stroke imaging protocol, to further support methodological reproducibility.

### 2.4 Image preprocessing and lesion segmentation

All MRI data underwent preprocessing including motion correction, spatial normalization, and intensity standardization using the SimpleITK framework. Infarct core regions were identified on ADC maps using a threshold of ADC < 600 × 10^−6^ mm^2^/s. Lesion segmentation was performed using semi-automated segmentation software integrated with a deep-learning–based stroke analysis pipeline. The pipeline used a convolutional neural network (U-Net-type architecture) trained to delineate acute ischemic lesions on DWI and ADC maps, implemented within the nnU-Net self-configuring segmentation framework (Isensee et al., 2021), trained on our institutional stroke MRI dataset with manually annotated infarct masks as ground truth. Segmented infarct masks were independently reviewed and corrected by two experienced neuroradiologists who were blinded to clinical outcomes. Inter-observer agreement between the two reviewing neuroradiologists for infarct mask delineation was assessed using the Dice similarity coefficient, which averaged 0.87 ± 0.05 (range, 0.78–0.94), indicating high concordance in lesion boundary delineation. Volumetric agreement was additionally confirmed using the intraclass correlation coefficient (ICC) for infarct lesion volume between the two raters (ICC = 0.93, 95% CI: 0.90–0.95), supporting high consistency of the segmentation-derived volumetric measurements used in subsequent analyses. Perfusion deficits were quantified using automated thresholding of Tmax maps with delay thresholds greater than 6 s. Perfusion mismatch volumes were calculated by subtracting diffusion lesion volume from the perfusion abnormality volume.

### 2.5 Radiomic feature extraction

Quantitative radiomic features were extracted from infarct regions on DWI, ADC, and perfusion maps using the PyRadiomics platform. Feature categories included first-order intensity statistics, shape descriptors, and higher-order texture features derived from gray-level co-occurrence matrix (GLCM), gray-level run-length matrix (GLRLM), gray-level size zone matrix (GLSZM), and neighboring gray tone difference matrix (NGTDM). Wavelet and Laplacian-of-Gaussian filters were applied to capture multiscale spatial heterogeneity within ischemic lesions. A total of 148 candidate radiomic features were initially extracted for each patient. Prior to model development, all radiomic features were standardized using z-score normalization to ensure comparability across variables with different scales. Features were centered to a mean of zero and scaled to a standard deviation of one. This preprocessing step was performed using the Scikit-learn preprocessing library to improve stability of machine learning algorithms and reduce bias related to feature magnitude. To prevent information leakage, the mean and standard deviation used for normalization were derived exclusively from the training cohort (defined in the Development of Predictive Models subsection below) and were then applied, without recalculation, to the testing cohort.

### 2.6 Feature selection and dimensionality reduction

To avoid information leakage, the dataset was first randomly partitioned into a training cohort (70%) and a testing cohort (30%), as described in the Development of Predictive Models subsection below. All steps described in this subsection, including removal of low-variance features, correlation-based redundancy filtering, and LASSO-based feature selection with 10-fold cross-validation, were performed using the training cohort only; the testing cohort was withheld from all feature-selection procedures and was not examined until the final, single evaluation of model performance.

To reduce feature redundancy and avoid model overfitting, a multistep feature selection strategy was applied. First, features with low variance were removed. Spearman correlation analysis was then used to eliminate highly correlated variables with correlation coefficients greater than 0.9. Finally, Least Absolute Shrinkage and Selection Operator (LASSO) regression with 10-fold cross-validation was used to select the most predictive imaging features for clinical outcomes.

### 2.7 Development of predictive models

Predictive models were constructed to estimate the risk of in-hospital mortality, hemorrhagic transformation, and poor functional outcome at 90 days. Clinical variables and selected radiomic features were integrated into several machine learning algorithms including logistic regression, random forest, support vector machine (SVM), and gradient boosting models. The dataset was randomly divided into a training cohort (70%) and a testing cohort (30%). Hyperparameters for each algorithm were optimized using grid search combined with repeated 10-fold cross-validation to improve generalizability. Because outcome events such as mortality and hemorrhagic transformation occurred less frequently than non-events, class imbalance was addressed using Synthetic Minority Oversampling Technique (SMOTE) applied within the training dataset. This approach generated synthetic samples of the minority class to balance the dataset and improve model sensitivity while preventing bias toward majority outcomes. Consistent with the feature-selection procedure above, SMOTE was applied strictly within the training folds. The held-out testing cohort was not used at any stage for feature selection, normalization-parameter estimation, or SMOTE-based resampling; it was reserved exclusively for a single, final evaluation of model performance.

### 2.8 Model performance and validation

Model performance was evaluated using receiver operating characteristic (ROC) analysis and calculation of the area under the curve (AUC). Additional metrics including sensitivity, specificity, accuracy, and F1 score were calculated to evaluate predictive performance. Calibration of predictive models was assessed using calibration plots and the Hosmer–Lemeshow goodness-of-fit test. Model performance was assessed using area under the receiver operating characteristic curve (AUC), sensitivity, specificity, accuracy, precision, recall, and F1-score. These metrics were calculated on the independent testing dataset to evaluate predictive discrimination and classification performance. Because the NIHSS is an ordinal scale with integer scoring, ROC-derived cut-points falling between consecutive integers (e.g., 8.5, 10.5) are intended to be applied clinically as the next higher whole-point score (e.g., a cut-point of ≥8.5 corresponds to NIHSS ≥9).

### 2.9 Explainability analysis

To improve model interpretability, Shapley Additive Explanation (SHAP) analysis was applied to quantify the contribution of individual features to model predictions. SHAP summary plots were generated to identify the most influential clinical and imaging variables associated with poor outcomes in AIS patients.

### 2.10 Feature importance analysis

Feature importance was evaluated using Shapley Additive Explanation (SHAP) values derived from the trained gradient boosting model. SHAP analysis quantifies the contribution of each feature to model predictions based on cooperative game theory principles. Global SHAP summary plots were used to identify the most influential radiomic and clinical predictors associated with poor outcomes. Feature importance rankings were generated based on mean absolute SHAP values.

### 2.11 Statistical analysis

Continuous variables were expressed as mean ± standard deviation or median with interquartile range depending on data distribution. Categorical variables were summarized as frequencies and percentages. Differences between groups were evaluated using Student's t-test or Mann–Whitney U test for continuous variables and chi-square tests for categorical variables. Multivariable logistic regression was used to identify independent predictors of clinical outcomes. Statistical analyses were conducted using R (version 4.2) and Python (Scikit-learn and PyRadiomics libraries), and a p-value < 0.05 was considered statistically significant. Optimal cut-off values for continuous clinical and imaging predictors were determined from ROC curve analysis using the Youden index (the threshold maximizing the sum of sensitivity and specificity), balancing correct classification of outcome and non-outcome cases within this cohort.

## 3 Results

Results are presented in order of increasing analytic complexity rather than in strict chronological order of analysis. Sections 3.1–3.7 report baseline characteristics and univariable and multivariable statistical associations between clinical and imaging variables and clinical outcomes, characterizing variable–outcome relationships across the full cohort. Sections 3.8–3.10 then report the performance of the machine learning models, including model selection, radiomics-based feature importance, and calibration analyses; these analyses were developed and evaluated using the training/testing partition, feature selection, class-imbalance correction, and cross-validation procedures detailed in the Methods (see Feature Selection and Dimensionality Reduction, Development of Predictive Models, and Model Performance and Validation). The gradient boosting model reported as selected in Section 3.8 was therefore chosen using this pre-specified analytic pipeline, independent of the univariable associations presented in the preceding sections.

### 3.1 Baseline characteristics of the study population

A total of 353 patients with acute ischemic stroke (AIS) were included in the analysis. The mean age of the study population was 63 ± 12 years, and 191 patients (54.1%) were male while 162 patients (45.9%) were female. Among patients with available data, hypertension was present in 178 of 315 patients (56.5%), diabetes mellitus in 60 of 320 (18.8%), and atrial fibrillation in 32 of 332 (9.6%). A history of smoking was reported in 129 of 315 patients (41.0%), and 30 of 324 patients (9.3%) had a prior history of stroke. The mean NIHSS score at admission was 8 ± 3, indicating moderate stroke severity. The average door-to-imaging time was 27 ± 10 min. Among the 310 patients with available data on vessel occlusion site, the most commonly involved vascular territory was the middle cerebral artery (MCA) in 140 patients (45.0%), followed by lacunar infarcts in 74 patients (23.8%), posterior cerebral artery in 43 patients (13.8%), internal carotid artery in 31 patients (10.0%), anterior cerebral artery in 9 patients (2.9%), basilar artery in 8 patients (2.6%), and vertebral artery in 5 patients (1.6%) (Table 1). Over the course of the study, in-hospital mortality occurred in 38 (10.8%) patients, hemorrhagic transformation in 81 (22.9%) patients, and recurrent ischemic stroke within 90 days in 20 (5.7%) patients (Table 1).

**Table 1:** Baseline characteristics of the study population (*N* = 353).

Variable	Value
Age (years), mean ± SD	63 ± 12
Sex, n (%)	
Male	191 (54.1)
Female	162 (45.9)
Hypertension, n (%)	
Yes	178 (56.5)
No	137 (43.5)
Diabetes mellitus, n (%)	
Yes	60 (18.8)
No	260 (81.3)
Atrial fibrillation, n (%)	
Yes	32 (9.6)
No	300 (90.4)
Smoking status, n (%)	
Yes	129 (41.0)
No	186 (59.0)
History of stroke, n (%)	
Yes	30 (9.3)
No	294 (90.7)
NIHSS score on admission, mean ± SD	8 ± 3
Door-to-imaging time (min), mean ± SD	27 ± 10
Vessel Occlusion Site, n (%)	
Middle cerebral artery	140 (45.0)
Lacunar infarcts	74 (23.8)
Posterior cerebral artery	43 (13.8)
Internal carotid artery	31 (10.0)
Anterior cerebral artery	9 (2.9)
Basilar artery	8 (2.6)
Vertebral artery	5 (1.6)
Clinical outcomes, *n* (%)	
In-hospital mortality	38 (10.8)
Hemorrhagic transformation	81 (22.9)
Recurrent ischemic stroke (90 days)	20 (5.7)

Percentages for hypertension, diabetes mellitus, atrial fibrillation, smoking status, history of stroke, and vessel occlusion site were calculated using the number of patients with available data for each variable as the denominator, which varies because of missing data: hypertension, *n* = 315; diabetes mellitus, *n* = 320; atrial fibrillation, *n* = 332; smoking status, *n* = 315; history of stroke, *n* = 324; vessel occlusion site, *n* = 310. All other percentages are based on *N* = 353.

### 3.2 Correlation between MRI parameters and clinical outcomes

Several MRI parameters demonstrated significant correlations with clinical outcomes (Table 2). Admission NIHSS score showed strong positive correlations with in-hospital mortality (ρ = 0.513, *p* < 0.001), hemorrhagic transformation (ρ = 0.662, *p* < 0.001), mRS score at discharge (ρ = 0.895, *p* < 0.001), and mRS score at 90 days (ρ = 0.803, *p* < 0.001). DWI lesion volume also correlated significantly with mortality (ρ = 0.518, *p* < 0.001), hemorrhagic transformation (ρ = 0.433, *p* < 0.001), and functional disability at discharge (ρ = 0.595, *p* < 0.001) and at 90 days (ρ = 0.620, *p* < 0.001).

**Table 2:** Correlation between MRI parameters and clinical outcomes.

Variable	Mortality (ρ)	Hemorrhage (ρ)	Recurrence (ρ)	mRS discharge (ρ)	mRS 90 days (ρ)
History of stroke	0.466^***^	0.398^***^	0.122^*^	0.367^***^	0.367^***^
NIHSS score	0.513^***^	0.662^***^	0.232^***^	0.895^***^	0.803^***^
DWI lesion volume	0.518^***^	0.433^***^	0.068	0.595^***^	0.620^***^
ADC minimum	−0.475^***^	−0.530^***^	−0.138^*^	−0.724^***^	−0.649^***^
Tmax	0.488^***^	0.574^***^	0.190^**^	0.777^***^	0.680^***^
CBF	−0.504^***^	−0.606^***^	−0.218^***^	−0.831^***^	−0.757^***^
CBV	−0.563^***^	−0.704^***^	−0.195^***^	−0.702^***^	−0.718^***^
MTT	0.530^***^	0.663^***^	0.215^***^	0.799^***^	0.709^***^
SWI microbleeds	0.497^***^	0.569^***^	0.174^**^	0.756^***^	0.708^***^
FLAIR hyperintensity	0.019	0.122^*^	−0.045	0.063	0.003
T2 lesion	0.067	0.078	−0.042	0.251^***^	0.155^*^
DWI-FLAIR mismatch	−0.030	−0.113^*^	0.012	−0.070	−0.003

^*^p < *0.05*, ^**^p < *0.01*,^***^p < *0.001*.

Lower ADC minimum values were inversely associated with poor outcomes, showing correlations with mortality (ρ = −0.475, *p* < 0.001), hemorrhagic transformation (ρ = −0.530, *p* < 0.001), and functional disability at discharge (ρ = −0.724, *p* < 0.001) and at 90 days (ρ = −0.649, *p* < 0.001). Perfusion parameters demonstrated similarly strong associations with outcomes. Prolonged Tmax correlated with mortality (ρ = 0.488, *p* < 0.001) and hemorrhagic transformation (ρ = 0.574, *p* < 0.001). Reduced cerebral blood flow showed strong negative correlations with disability at discharge (ρ = −0.831, *p* < 0.001) and at 90 days (ρ = −0.757, *p* < 0.001). Mean transit time exhibited strong correlations with mRS at discharge (ρ = 0.799, *p* < 0.001) and at 90 days (ρ = 0.709, *p* < 0.001). SWI microbleed count also demonstrated strong correlations with adverse outcomes including mortality (ρ = 0.497, *p* < 0.001) and functional disability at discharge (ρ = 0.756, *p* < 0.001). In contrast, FLAIR hyperintensity and T2 hyperintense lesions showed weaker or non-significant correlations with most clinical outcomes (Table 2).

### 3.3 Prediction of in-hospital mortality

Receiver operating characteristic (ROC) analysis demonstrated excellent predictive performance for several clinical and imaging variables (Supplementary Table 1). The NIHSS score at admission showed strong discrimination with an AUC of 0.963, sensitivity of 94.29%, specificity of 90.97%, and overall accuracy of 91.35% using a threshold of ≥10.5. DWI lesion volume demonstrated similarly high predictive performance with an AUC of 0.975, sensitivity of 94.12%, specificity of 92.62%, and accuracy of 92.79% at a cut-off value of ≥52.5 mL. Cerebral blood flow ≤ 20.5 showed excellent predictive ability with an AUC of 0.975, sensitivity of 100%, and specificity of 90.49%. Additional predictors included ADC minimum (AUC 0.951, sensitivity 96.43%, specificity 88.93%), Tmax delay ≥8.2 s (AUC 0.952, sensitivity 87.88%, specificity 85.77%), mean transit time ≥8.5 s (AUC 0.973, sensitivity 100%, specificity 87.59%), and SWI microbleed count ≥6.5 (AUC 0.968, sensitivity 96.77%, specificity 85.50%). Negative predictive values were extremely high across these parameters, reaching 99–100%, indicating strong ability to rule out mortality risk (Supplementary Table 1; Figure 1).

**Figure 1:**
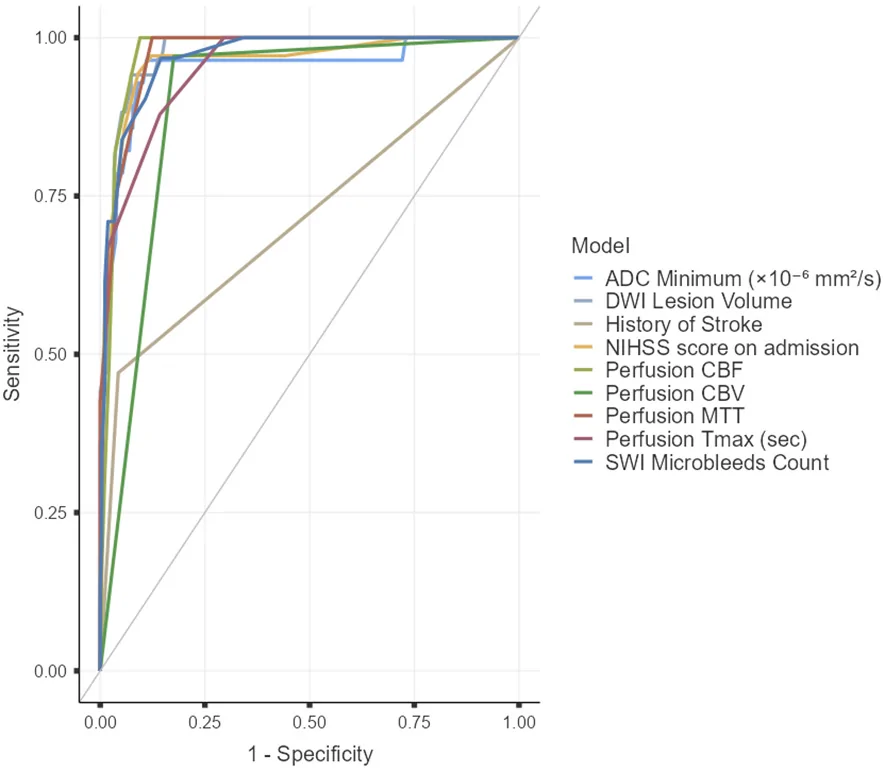
In-hospital mortality predictors: receiver operating characteristic (ROC) curves comparing individual clinical and imaging predictors (NIHSS score on admission, DWI lesion volume, ADC minimum, perfusion CBF, CBV, MTT, and Tmax, SWI microbleed count, and history of stroke) for discriminating patients who died in-hospital from survivors; each colored curve corresponds to one predictor as indicated in the legend, and the diagonal gray line denotes the line of no discrimination (AUC = 0.50).

### 3.4 Prediction of hemorrhagic transformation

Hemorrhagic transformation was also well predicted by several imaging parameters (Supplementary Table 2). The NIHSS score at admission demonstrated strong predictive performance with an AUC of 0.948, sensitivity of 88.41%, specificity of 89.61%, and overall accuracy of 89.33% at a threshold of ≥8.5. Mean transit time ≥8.5 s showed excellent discrimination with an AUC of 0.931, sensitivity of 74.29%, and specificity of 94.64%. Perfusion Tmax ≥7.5 s demonstrated an AUC of 0.894 with sensitivity of 86.36% and specificity of 77.35%.

Other significant predictors included cerebral blood flow ≤ 22.5 (AUC 0.910), cerebral blood volume ≤ 1.5 (AUC 0.871), ADC minimum ≤ 447.5 (AUC 0.867) (Liu et al., 2022), and SWI microbleed count ≥6.5 (AUC 0.894) . In contrast, FLAIR hyperintensity showed poor predictive performance with an AUC of 0.568 (Supplementary Table 2; Figure 2).

**Figure 2:**
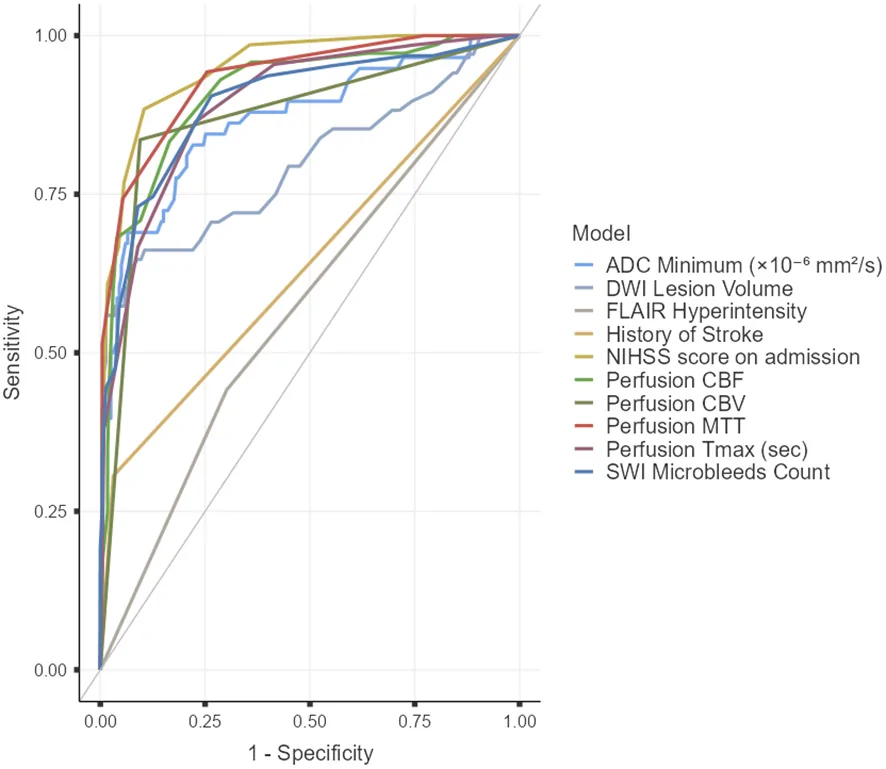
Haemorrhagic transformation predictors: receiver operating characteristic (ROC) curves comparing individual clinical and imaging predictors (NIHSS score on admission, DWI lesion volume, ADC minimum, FLAIR hyperintensity, perfusion CBF, CBV, MTT, and Tmax, and SWI microbleed count) for discriminating patients who developed hemorrhagic transformation from those who did not; curve colors correspond to the legend, and the diagonal gray line denotes the line of no discrimination (AUC = 0.50).

### 3.5 Prediction of recurrent stroke within 90 days

Prediction of recurrent stroke within 90 days demonstrated moderate discrimination (Supplementary Table 3). Admission NIHSS score ≥8.5 produced the highest predictive performance with an AUC of 0.786, sensitivity of 81.25%, specificity of 73.23%, and accuracy of 73.68%. Perfusion parameters also demonstrated moderate predictive value. Tmax ≥6.5 s showed an AUC of 0.735 with sensitivity of 93.75%, while CBF ≤ 23.5 produced an AUC of 0.767 with sensitivity of 94.12%. CBV ≤ 1.5 demonstrated an AUC of 0.700, and MTT ≥7.5 showed an AUC of 0.766. SWI microbleed count ≥5.5 produced an AUC of 0.713. Across these predictors, negative predictive values were consistently high (96–99%), indicating strong ability to exclude recurrence risk, whereas positive predictive values remained relatively low (9–15%) (Supplementary Table 3; Figure 3).

**Figure 3:**
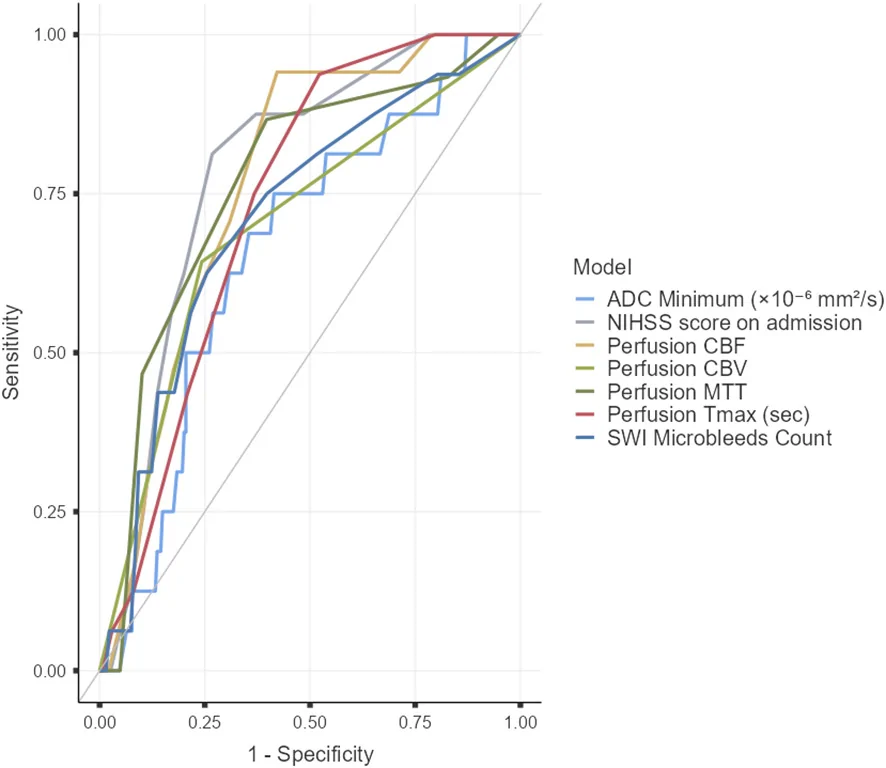
Recurrent Stroke within 90 Days predictors: receiver operating characteristic (ROC) curves comparing individual clinical and imaging predictors (NIHSS score on admission, ADC minimum, perfusion CBF, CBV, MTT, and Tmax, and SWI microbleed count) for discriminating patients who experienced recurrent ischemic stroke within 90 days from those who did not; curve colors correspond to the legend, and the diagonal gray line denotes the line of no discrimination (AUC = 0.50).

### 3.6 Predictors of functional disability

Univariate regression analysis identified several clinical and imaging variables significantly associated with functional disability. Hypertension was associated with increased mRS score at discharge (β = 0.50 ± 0.21, *p* = 0.019) and at 90 days (β = 0.64 ± 0.25, *p* = 0.010). Diabetes mellitus was also associated with worse outcomes at discharge (β = 0.98 ± 0.26, *p* < 0.001) and at 90 days (β = 0.95 ± 0.31, *p* = 0.002). History of prior stroke demonstrated the strongest association with disability, with regression estimates of β = 2.53 ± 0.30 (*p* < 0.001) at discharge and β = 3.07 ± 0.33 (*p* < 0.001) at 90 days. Admission NIHSS score was strongly associated with worse outcomes (β = 0.45 ± 0.01, *p* < 0.001 at discharge; β = 0.47 ± 0.02, *p* < 0.001 at 90 days). Imaging parameters including larger DWI lesion volume, prolonged Tmax and MTT, reduced cerebral blood flow and cerebral blood volume, and increased SWI microbleed count were all significantly associated with higher mRS scores at both time points (all p < 0.001) (Table 3).

**Table 3:** Univariate regression analysis for functional outcomes.

Predictor	Discharge β	*SE*	*p*	90-day β	*SE*	*p*
Hypertension	0.50	0.21	0.019	0.64	0.25	0.010
Diabetes mellitus	0.98	0.26	< 0.001	0.95	0.31	0.002
History of stroke	2.53	0.30	< 0.001	3.07	0.33	< 0.001
NIHSS	0.45	0.01	< 0.001	0.47	0.02	< 0.001
DWI lesion volume	0.06	0.004	< 0.001	0.08	0.004	< 0.001
ADC minimum	−0.02	0.001	< 0.001	−0.022	0.002	< 0.001
Tmax	0.75	0.04	< 0.001	0.75	0.05	< 0.001
CBF	−0.31	0.01	< 0.001	−0.30	0.02	< 0.001
CBV	−2.73	0.15	< 0.001	−3.02	0.18	< 0.001
MTT	0.99	0.04	< 0.001	1.03	0.05	< 0.001
SWI microbleeds	0.30	0.01	< 0.001	0.32	0.014	< 0.001

### 3.7 Multivariable logistic regression for independent predictors of mortality

To identify independent predictors of in-hospital mortality, variables that were significant in univariate analyses were entered into a multivariable logistic regression model. Admission NIHSS score, DWI lesion volume, cerebral blood flow (CBF), mean transit time (MTT), and SWI microbleed count remained independent predictors of mortality. Higher NIHSS score and larger infarct volume were associated with increased mortality risk, while higher CBF was associated with lower mortality risk. Perfusion delay represented by prolonged MTT also significantly increased mortality risk (Table 4).

**Table 4:** Multivariable logistic regression predicting in-hospital mortality.

Predictor	Odds ratio (OR)	95% CI	*p*-value
NIHSS score	1.31	1.21–1.42	< 0.001
DWI lesion volume (per mL)	1.04	1.02–1.06	< 0.001
CBF	0.91	0.88–0.95	< 0.001
MTT	1.22	1.11–1.34	< 0.001
SWI microbleeds	1.18	1.07–1.30	0.001

### 3.8 Machine learning predictive modeling

Machine-learning models were developed to improve prediction of mortality and poor functional outcomes. Clinical and imaging variables were used as input features for logistic regression, random forest, support vector machine (SVM), and gradient boosting models. The dataset was randomly divided into training (70%) and testing (30%) cohorts, and hyperparameters were optimized using 10-fold cross-validation. Among the tested algorithms, the gradient boosting model demonstrated the highest predictive performance. The gradient boosting model significantly improved discrimination compared with logistic regression alone (Table 5).

**Table 5:** Machine learning model performance for mortality prediction.

Model	AUC	Sensitivity (%)	Specificity (%)	Accuracy (%)
Logistic regression	0.91	88.1	85.4	86.5
Random forest	0.94	90.2	88.6	89.4
Support vector machine	0.92	89.0	86.7	87.5
Gradient boosting	**0.96**	**93.4**	**91.2**	**92.5**

Bold values indicate the best-performing result for each metric among the evaluated models.

### 3.9 Radiomics feature importance analysis

Radiomic features were extracted from diffusion and perfusion images to quantify lesion heterogeneity. Feature selection using LASSO regression reduced the initial set of 148 radiomic variables to the most predictive imaging biomarkers. Feature importance was further evaluated using SHAP analysis (Table 6; Figure 4), which demonstrated that imaging heterogeneity and diffusion restriction were strong predictors of adverse outcomes. The selected hyperparameters for the gradient boosting and support vector machine models are summarized in Table 7.

**Table 6:** Top radiomic features associated with poor outcome.

Radiomic feature	Importance score
ADC entropy	0.18
Perfusion heterogeneity (GLCM)	0.16
Wavelet-filtered Tmax texture	0.14
DWI lesion irregularity	0.13
ADC kurtosis	0.11
Perfusion intensity variance	0.10

**Figure 4:**
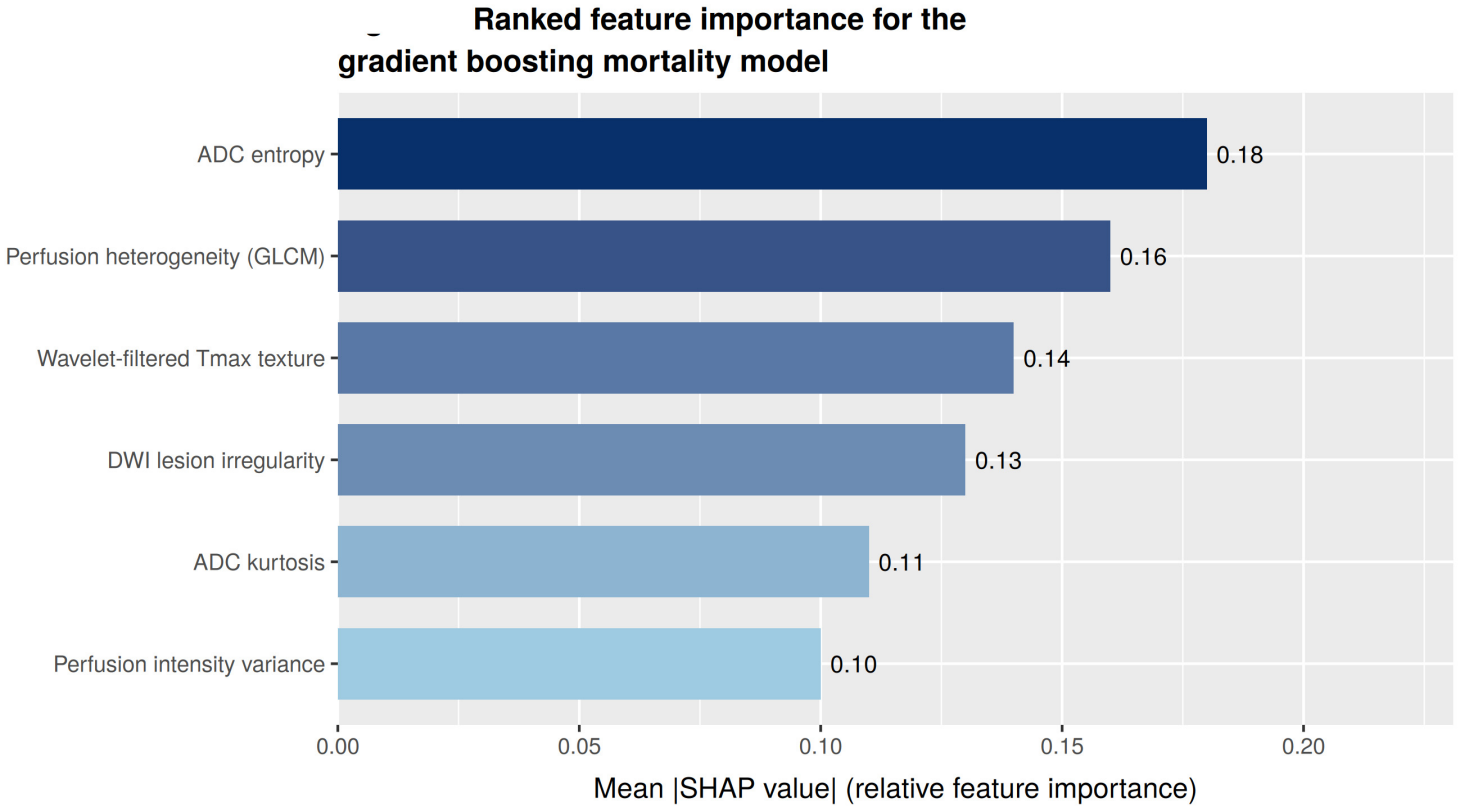
SHAP summary plot illustrating the relative contribution of clinical and radiomic features (ranked by mean absolute SHAP value) to gradient boosting model predictions of in-hospital mortality, corresponding to the features ranked in Table 6.

**Table 7:** Selected hyperparameters for the gradient boosting and support vector machine models.

Model	Hyperparameter	Selected value
Gradient boosting	Number of estimators	300
Gradient boosting	Learning rate	0.05
Gradient boosting	Maximum tree depth	4
Gradient boosting	Subsample ratio	0.8
Support vector machine	Kernel	Radial basis function (RBF)
Support vector machine	C (regularization)	1.0
Support vector machine	Gamma	0.01

Values were selected via grid search combined with repeated 10-fold cross-validation within the training cohort only, as described in the Methods (Development of Predictive Models).

### 3.10 Model calibration and clinical utility

Calibration analysis demonstrated strong agreement between predicted and observed outcomes, with a Hosmer–Lemeshow *p*-value of 0.42, indicating good model calibration. Decision curve analysis showed that the machine-learning models provided greater net clinical benefit across threshold probabilities between 10% and 70%, supporting the clinical utility of integrating multiparametric MRI biomarkers into prognostic assessment.

## 4 Discussion

In this study, we found that multiparametric MRI provides powerful prognostic value in AIS cases. The analysis of 353 cases diagnosed with AIS showed significant association between larger diffusion lesions and more severe perfusion deficits with poor clinical outcomes, which is consistent with established evidence about infarct core size and perfusion deficits and their correlation with clinical outcomes in stroke patients (Nannoni et al., 2021). In particular, admission NIHSS, DWI lesion volume, Tmax and MTT delays, and SWI microbleed burden each showed strong positive correlations with in-hospital mortality and functional disability, whereas higher ADC, CBF, and CBV correlated inversely with poor outcomes. For instance, discharge mRS scores strongly correlated with NIHSS scores (ρ = 0.895) and with prolonged perfusion times (MTT ρ = 0.799, Tmax ρ = 0.777), while ADC minimum, CBF, and CBV showed similarly robust inverse correlations. These findings are consistent with previous studies by Chalos et al. and Banks et al., who reported that NIHSS at 24 h and 7 days of admission had significant correlation with mRS during follow-up in patients with AIS (Chalos et al., 2020; Banks and Marotta, 2007).

Mechanistically, these correlations likely reflect the combined contribution of infarct core size and hypoperfused but potentially salvageable tissue (the ischemic penumbra) to clinical severity: larger DWI lesions and lower ADC values indicate more extensive irreversible tissue injury, while prolonged MTT and Tmax reflect the degree of impaired collateral perfusion, both established determinants of neurological deterioration and functional recovery after AIS. The strength of these associations in our cohort, several of which exceeded those reported in smaller single-modality studies, may reflect the added discriminatory value of combining diffusion, perfusion, and susceptibility-based markers within a single multiparametric framework rather than relying on any single sequence.

In multivariate analyses of functional disability, admission NIHSS and prior stroke were strong predictors for clinical prognosis. Similarly, imaging parameters also independently predicted clinical outcomes in AIS patients since larger DWI volume and longer MTT, were positively associated with higher mRS scores. These correlations highlight that patients with extensive infarction and delayed perfusion tend to have worse functional outcomes, shown by higher mRS scores. In contrast, lower ADC minimum (restricted diffusion), CBF, and CBV showed inverse association with mRS at both discharge and after 90 days, indicating higher functional disability. These findings are consistent with a previous study by Li et al., who reported higher CBF and CBV (r = −0.459 and −0.502, respectively) were associated with a lower 90-day mRS with better functional outcomes (Li et al., 2024).

These imaging–clinical relationships translated into highly discriminatory prognostic thresholds in AIS cases. Admission NIHSS ≥10.5 and DWI lesion volume ≥52.5 mL showed AUCs of 0.963 and 0.975 and accuracies of 91.35% and 92.79%, respectively; CBF ≤ 20.5 showed an AUC of 0.975. Additionally, prolonged perfusion delays (MTT ≥8.5 s, Tmax ≥8.2 s) and very low ADC correctly predicted mortality in over 95% of cases. Notably, an SWI microbleed count ≥6.5 had an AUC of 0.968 and a sensitivity of 96.77% for in-hospital mortality. For hemorrhagic transformation, an NIHSS score ≥8.5 had an AUC of 0.948 and an accuracy of 89.33%, and an MTT ≥8.5 s had an AUC of 0.931, a sensitivity of 74.29%, and a specificity of 94.64%. This aligns with Cucchiara et al. (2008), who identified NIHSS as a good predictor of intracranial hemorrhage in AIS patients.

Regarding CBF, Tmax, and CBV, ADC minimum thresholds showed good, but less predictive values compared to NIHSS scores with accuracy ranging between 86.7 to 91%, unlike FLAIR hyperintensity, which revealed poor prediction for hemorrhagic transformation in only 56.8% of cases who experienced parenchymal hemorrhage. Tong et al. noticed that regions with very low ADC on acute DWI tended to develop hemorrhage and that persistent perfusion deficits strongly predicted secondary hemorrhagic transformation (Tong et al., 2001). Similarly, Nael et al. reported that CBV independently predicted development of parenchymal hemorrhage in patients with AIS (Nael et al., 2017). These insights are consistent with our results, indicating that the identification of specific MRI cut-offs could significantly stratify patients by risk of death, hemorrhage, and disability.

On predicting the risk of recurrence, NIHSS scores higher than 8.5 points at admission could correctly distinguish recurrence vs. no recurrence of stroke after 90 days in about 78.6% of cases, indicating moderate discriminative ability. Similarly, perfusion thresholds demonstrated modest AUC values ranging from 0.70 to 0.77 with very high negative predictive values between 97-99%, suggesting that patients below these thresholds are unlikely to experience recurrence, which helps in ruling out recurrence in such cases; however, low positive predictive values indicate limited utility for confirming recurrence risk. This dissociation reflects the low base-rate (prevalence) of 90-day recurrence in our cohort (20/353 patients, 5.7%) rather than poor discriminative ability of the perfusion parameters themselves. Because positive predictive value depends jointly on sensitivity, specificity, and outcome prevalence, applying Bayes' theorem to, for example, the CBF ≤ 23.5 threshold (sensitivity 94.1%, specificity 57.8%) at this prevalence yields an expected PPV of approximately 12%, closely matching the empirically observed range (9–15%). This confirms that the low PPV is a mathematical consequence of predicting a rare event rather than a limitation specific to these imaging parameters: in low-prevalence settings, even sensitive tests inevitably generate more false positives than true positives in absolute numbers, and PPV improves only when applied to enriched, higher-risk subpopulations. Juli et al., reported that NIHSS scores correlated with recurrent stroke, but in this study we determined NIHSS threshold for recurrence risk with predictive values, which enhance clinical practice (Juli et al., 2022).

Beyond these threshold-based imaging metrics, our radiomics analyses add a complementary, higher-dimensional perspective to prognostication in AIS. The texture and heterogeneity features identified by SHAP analysis (Section 3.9) suggest that microstructural heterogeneity within the infarct core and surrounding perfusion deficit, captured only indirectly by conventional volumetric or threshold-based measures, may carry additional prognostic information. This is consistent with Yang et al., who reviewed the expanding application of radiomics across the diagnostic and prognostic spectrum of ischemic stroke, highlighting its potential to capture subtle tissue-level heterogeneity beyond visual inspection (Yang et al., 2024). Similarly, Tolhuisen et al. demonstrated that automatically extracted infarct core image features improved prediction of functional outcome after AIS beyond clinical variables alone (Tolhuisen et al., 2022). Together with these reports, our findings support the view that radiomic feature analysis, particularly when combined with clinical and conventional imaging variables as in the present study, can meaningfully augment outcome prediction in AIS; further work directly comparing the incremental value of radiomic features over conventional thresholds, ideally in externally validated cohorts, is warranted.

We acknowledge that the individual machine learning algorithms applied here (logistic regression, random forest, support vector machine, and gradient boosting) are well-established rather than novel in themselves. However, we would argue that the principal contribution of this work lies less in algorithmic novelty and more in translational integration: to our knowledge, this is among the first studies to combine multiparametric MRI radiomics, explainable AI (SHAP-based feature attribution), and clinically interpretable, outcome-specific thresholds across four distinct endpoints – mortality, hemorrhagic transformation, 90-day recurrence, and functional disability – within a single, rigorously leakage-controlled analytic pipeline. This comprehensive, multi-endpoint framework, together with the explicit quantitative thresholds derived for bedside use, differentiates our approach from prior single-outcome or single-modality studies and was designed with clinical usability, rather than methodological novelty *per se*, as the primary objective.

### 4.1 Clinical implications

In clinical practice, MRI-derived thresholds can enhance risk stratification, through identification of patients with very large cores or severe perfusion delay on admission to provide early aggressive care, such as intensive monitoring, blood pressure control, early rehabilitation for high-risk groups to avoid or at least decrease risk of complications. Additionally, multiparametric MRI findings may help in decision-making for management since perfusion and diffusion imaging is already used to select thrombectomy candidates in extended time windows (Powers et al., 2019). Thus, knowing that certain thresholds predict hemorrhage, including very low CBF and high Tmax might influence the risk–benefit assessment for aggressive reperfusion therapy. Moreover, these quantitative MRI metrics provide objective data to discuss expected outcomes with patients and families about prognosis prediction. For instance, a patient with DWI volume over 50 mL and multiple microbleeds can be counseled about the high likelihood of severe disability or mortality. Finally, the high negative predictive values for recurrence suggest MRI could help rule out short-term recurrence risk for secondary prevention strategies or outpatient follow-up. Given their low positive predictive value, these recurrence thresholds are best applied as a triage step to identify patients at low risk who may be reassured and followed as outpatients, whereas patients exceeding these thresholds should undergo further confirmatory clinical, laboratory, or vascular imaging assessment rather than being classified as high-risk for recurrence on the basis of the MRI threshold alone.

### 4.2 Limitations

Although our study involved 353 AIS patients, which is a relatively large sample size compared to prior MRI studies (Nael et al., 2017; Nannoni et al., 2021), the findings from this study should be interpreted carefully since many associations and cut-points were derived from the same cohort without external validation, raising the risk of overfitting and limiting generalizability. The predictive modeling relied largely on univariate correlations, regressions and ROC-based thresholds, which do not fully adjust for confounding or interaction among covariates. Finally, positive predictive values for 90-day recurrence were low despite high negative predictive values. Therefore, those recurrence thresholds are better suited for exclusionary screening rather than confirmation and should not be used alone for clinical decision-making. A further limitation concerns the rigor of model validation: although hyperparameter tuning and feature selection were confined to the training cohort as described in the Methods, model performance was assessed using a single train/test partition rather than nested cross-validation, and no independent external cohort was available. The reported performance metrics should therefore be interpreted as reflecting internal, within-cohort discrimination, informative for comparing candidate models during development, rather than as unbiased estimates of real-world predictive performance. Future work incorporating nested cross-validation and, critically, evaluation in independent external cohorts is needed before these models or thresholds can be considered for clinical deployment. While a full external validation was beyond the scope of the present retrospective analysis, we agree this is an important next step. We plan to evaluate the reproducibility of the identified radiomic features and threshold-based classifiers using publicly available stroke imaging repositories with linked outcome data (e.g., the ISLES 2022 acute ischemic stroke lesion segmentation challenge dataset), extended with prospectively collected clinical follow-up, as a priority for future work. We note, however, that most existing public stroke MRI datasets provide imaging data without linked longitudinal clinical outcomes such as 90-day recurrence or mortality, which limited the feasibility of a fully externally validated analysis within the current revision timeline.

## 5 Conclusion

In conclusion, Multiparametric MRI demonstrated strong prognostic value in AIS by accurately correlating diffusion and perfusion abnormalities with mortality, hemorrhagic transformation, and functional disability. Quantitative MRI thresholds such as DWI lesion volume, CBF, and MTT effectively predicted outcomes, outperforming conventional imaging. These findings support further external validation of multiparametric MRI for early risk stratification and individualized management.

## Data Availability

The raw data supporting the conclusions of this article will be made available by the authors, without undue reservation.

## Supplementary material

Supplementary Figure 1 *I*llustration of multiparametric MRI in acute ischemic stroke demonstrating a left MCA infarct with diffusion restriction on DWI corresponding ADC reduction a larger left MCA Tmax perfusion deficit and a susceptibility-blooming microbleed on SWI (arrow).

Supplementary Table 1 Predictors of in-hospital mortality (ROC analysis).

Supplementary Table 2 Predictors of hemorrhagic transformation.

Supplementary Table 3 Predictors of recurrent stroke within 90 days.
