# Supplementary material for: Multiparametric MRI radiomics and machine learning for outcome prediction in acute ischemic stroke: a retrospective study of 353 patients
Source: Front Neurosci. 2026 Oct 22;20:1843917. doi: 10.3389/fnins.2026.1843917 (PMC13736720; doi:10.3389/fnins.2026.1843917)
Supplement: Supplementary file 1 [file Table_1.docx]

# **Table S1. Predictors of In-Hospital Mortality (ROC Analysis)**

| **Predictor** | **Cut-off** | **AUC** | **SE** | **Sensitivity (%)** | **Specificity (%)** | **PPV** | **NPV** | **Accuracy (%)** |
| --- | --- | --- | --- | --- | --- | --- | --- | --- |
| **History of stroke** | **≥0.5** | **0.714** | **0.044** | **47.06** | **95.68** | **57.14** | **93.66** | **90.38** |
| **NIHSS** | **≥10.5** | **0.963** | **0.018** | **94.29** | **90.97** | **56.9** | **99.21** | **91.35** |
| **DWI lesion volume** | **≥52.5** | **0.975** | **0.008** | **94.12** | **92.62** | **61.54** | **99.21** | **92.79** |
| **ADC minimum** | **≤435** | **0.951** | **0.026** | **96.43** | **88.93** | **50.0** | **99.54** | **89.71** |
| **Tmax** | **≥8.2** | **0.952** | **0.013** | **87.88** | **85.77** | **42.03** | **98.37** | **85.99** |
| **CBF** | **≤20.5** | **0.975** | **0.008** | **100** | **90.49** | **55.0** | **100** | **91.48** |
| **CBV** | **≤1.5** | **0.897** | **0.019** | **97.06** | **82.44** | **40.24** | **99.57** | **84.03** |
| **MTT** | **≥8.5** | **0.973** | **0.008** | **100** | **87.59** | **49.25** | **100** | **88.93** |
| **SWI microbleeds** | **≥6.5** | **0.968** | **0.011** | **96.77** | **85.50** | **43.48** | **99.57** | **86.67** |

# **Table S2. Predictors of Hemorrhagic Transformation**

| **Predictor** | **Cut-off** | **AUC** | **Sensitivity (%)** | **Specificity (%)** | **Accuracy (%)** |
| --- | --- | --- | --- | --- | --- |
| **History of stroke** | **≥0.5** | **0.637** | **30.56** | **96.94** | **81.06** |
| **NIHSS** | **≥8.5** | **0.948** | **88.41** | **89.61** | **89.33** |
| **DWI lesion volume** | **≥44.5** | **0.797** | **64.71** | **91.23** | **85.14** |
| **ADC minimum** | **≤447.5** | **0.867** | **68.97** | **93.50** | **87.98** |
| **Tmax** | **≥7.5** | **0.894** | **86.36** | **77.35** | **79.33** |
| **CBF** | **≤22.5** | **0.910** | **83.33** | **83.48** | **83.44** |
| **CBV** | **≤1.5** | **0.871** | **83.58** | **90.52** | **88.96** |
| **MTT** | **≥8.5** | **0.931** | **74.29** | **94.64** | **89.80** |
| **SWI microbleeds** | **≥6.5** | **0.894** | **73.02** | **91.03** | **87.06** |
| **FLAIR hyperintensity** | **≥0.5** | **0.568** | **44.12** | **69.87** | **63.97** |

# **Table S3. Predictors of Recurrent Stroke Within 90 Days**

| **Predictor** | **Cut-off** | **AUC** | **Sensitivity (%)** | **Specificity (%)** | **Accuracy (%)** |
| --- | --- | --- | --- | --- | --- |
| **NIHSS** | **≥8.5** | **0.786** | **81.25** | **73.23** | **73.68** |
| **ADC minimum** | **≤488.5** | **0.662** | **75.00** | **58.55** | **59.60** |
| **Tmax** | **≥6.5** | **0.735** | **93.75** | **47.79** | **50.35** |
| **CBF** | **≤23.5** | **0.767** | **94.12** | **57.82** | **59.93** |
| **CBV** | **≤1.5** | **0.700** | **64.29** | **75.72** | **75.17** |
| **MTT** | **≥7.5** | **0.766** | **86.67** | **60.30** | **61.70** |
| **SWI microbleeds** | **≥5.5** | **0.713** | **62.50** | **74.52** | **73.82** |

*Note: The National Institutes of Health Stroke Scale (NIHSS) is an ordinal scale with integer scoring. Cut-points above that fall between consecutive integers (e.g., 8.5, 10.5) reflect the optimal ROC-derived threshold and are intended to be applied clinically as the next higher whole-point score (e.g., a cut-point of ≥8.5 corresponds to NIHSS ≥9). See the main manuscript, Model Performance and Validation, for further detail.*
